# Telemedicine and laryngology in Brazil: current situation, limitations, and prospects

**DOI:** 10.1016/j.bjorl.2021.04.003

**Published:** 2021-04-25

**Authors:** Gustavo Polacow Korn, Virgilio Prado, Vania Rosa Moraes

**Affiliations:** a Universidade Federal de São Paulo, São Paulo, SP, Brazil; b Ex-Presidente da Academia Brasileira de Laringologia e Voz (ABLV), São Paulo, SP, Brazil; c Associação Brasileira de Otorrinolaringologia e Cirurgia Cérvico-Facial (ABORL-CCF), Comitê de Defesa Profissional e da Comissão de Telemedicina, São Paulo, SP, Brazil; d Associação Brasileira de Otorrinolaringologia e Cirurgia Cérvico-Facial (ABORL-CCF), Departamento Jurídico, São Paulo, SP, Brazil

The COVID-19 pandemic has sped up the development and implementation of Telemedicine in Brazil. Given the ease of viral transmission, the recommendation to prevent direct contact between people led to an unprecedented change in health care. In Brazil, the government authorized remote medical assessment, on an exceptional basis, during the pandemic through federal law 13.989 and CFM Official Letter 1756/2020, which does not impose any specific restriction to its practice.^1^, ^2^ Hundreds of publications in several health areas in 2020 and 2021 show its feasibility. However, in the field of larynx and voice, several questions remain unanswered in the daily work of the laryngologist.

Aside from the technical limitations of remote medical assessment, such as the quality of internet connection, which would affect any other health area, reaching a diagnosis without static and dynamic evaluation of the larynx and vocal tract poses some possible hazards. Strohl et al.^3^ emphasize that this larynx and vocal tract evaluation is vital and should be conducted as soon as possible for safety reasons. Castillo-Allendes et al.,^4^ in a guideline for speech therapy, also reinforce the importance of carrying out this evaluation as quickly as possible.

There is a joint voice evaluation in laryngological centers by physicians and speech therapists; however, it is conducted only by the physician in some others. Auditory perceptual assessment of voice leads to understanding the vocal alteration, but this relation is not always present. In this context, auditory training for voice evaluation is essential once we do not have larynx evaluation, usually not always available in perfect conditions during remote assistance. In the daily practice of the laryngologist, endoscopic findings are not always accompanied by vocal alterations, as, for example, a marked supraglottic constriction in a patient with mild or discrete vocal tension.

Another topic that needs consideration is professional accountability. What happens in cases where the patient suffers misdiagnoses with a negative outcome?

A possible situation would be a singer with acute dysphonia due to vocal fold hemorrhage. After three weeks, the laryngologist may not observe signs of bleeding and thus diagnose it as functional dysphonia.

In 1999, the National Voice Campaign was launched, which then turned into a World event with the World Voice Day.^5^ In the campaign, a frequent emphasis is that every person with a vocal alteration lasting longer than two weeks should undergo a presential medical assessment. A thorough static and dynamic larynx and vocal tract evaluation are essential since a possible diagnosis is early larynx cancer which prompts detection offers the best chance of cure. Therefore, it is crucial to question the implications of postponing a complete medical evaluation when evaluation is remote. Also, the importance of assessing the patient in person as soon as possible is evident. Since there is no guarantee, this will occur sooner; physicians should always keep their permanent ethical and civil responsibility unaltered and emphatically recommend in-person evaluations even when symptoms improve.

Technical limitations for remote evaluation will lead to a non-diagnostic medical appointment. It will force physicians to interrupt assistance without determining the diagnosis and treatment. Then, it requires an in-person visit to their office. In this context, it is essential that limitations of telemedicine be previously informed and explained to patients and that this evaluation recorded in detail in their medical charts as a digital document. It ensures the continuity of health assistance and defines the rationale for treatment decisions.

One other raised question is whether patients are ready for a remote medical appointment. We lack research to confirm it, but the general impression is that patients still prefer in-person assistance. Those who seek for remote service do it for fear of SARS-COV-2 contagion, not for considering its reliability. It is essential to point out that up to the time we write this editorial, about 15% of the Brazilian population was already vaccinated (first dose).

Remote medical assistance did not begin during the current Covid-19 pandemic, but it strongly stimulated. Still, these services are likely to remain even with the much-desired return to the pre-pandemic routine. We anticipate that the more significant growth vector will be technological advancement to conduct remote physical exams, which is currently the main obstacle for reasonable Telemedicine in laryngology. Small physical exams should advance on a method-by-method basis, gradually supporting problem-solving decisions in remote medical care. We conceive the prospects more easily than well-defined. It is up to each one of us as specialist physicians to be attentive and critical of all innovations that have the power to change the way we conduct remote assistance in laryngology.

## Conflicts of interest

The authors declare no conflicts of interest.
